# Molecular analysis and epidemiological typing of Vancomycin-resistant *Enterococcus* outbreak strains

**DOI:** 10.1038/s41598-019-48436-2

**Published:** 2019-08-15

**Authors:** Anbjørg Rangberg, Astri Lervik Larsen, Oliver Kacelnik, Hanne Skarpodde Sæther, Marthe Bjørland, Jetmund Ringstad, Christine Monceyron Jonassen

**Affiliations:** 1 Center for Laboratory Medicine, Østfold Hospital Trust, Grålum, Norway; 2 Department of Antibiotic Resistance and Infection Prevention, Norwegian Institute of Public Health, Oslo, Norway; 3 Department of Infectious Diseases, Østfold Hospital Trust, Grålum, Norway; 4 Department of Chemistry, Biotechnology and Food Science, Norwegian University of Life Sciences, Ås, Norway

**Keywords:** Microbiology techniques, Clinical microbiology, Pathogens, Bacterial infection

## Abstract

Outbreaks of multidrug resistant bacteria including vancomycin-resistant enterococci (VRE) in healthcare institutions are increasing in Norway, despite a low level of resistance compared to other European countries. In this study, we describe epidemiological relatedness of vancomycin-resistant *Enterococcus faecium* isolated during an outbreak at a Norwegian hospital in 2012–2013. During the outbreak, 9454 fecal samples were screened for VRE by culture and/or PCR. Isolates from 86 patients carrying the *vanA* resistance gene were characterized using pulsed-field gel electrophoresis (PFGE), MALDI-TOF mass spectrometry and single nucleotide polymorphism typing. PFGE revealed two main clusters, the first comprised 56 isolates related to an initial outbreak strain, and the second comprised 21 isolates originating from a later introduced strain, together causing two partly overlapping outbreaks. Nine isolates, including the index case were not related to the two outbreak clusters. In conclusion, the epidemiological analyses show that the outbreak was discovered by coincidence, and that infection control measures were successful. All typing methods identified the two outbreak clusters, and the experiment congruence between the MALDI-TOF and the PFGE clustering was 63.2%, with a strong correlation (r = 72.4%). Despite lower resolution compared to PFGE, MALDI-TOF may provide an efficient mean for real-time monitoring spread of infection.

## Introduction

Enterococci are commensals of the gastrointestinal tract of humans and other mammals but are also a leading cause of healthcare associated infections^1^. *Enterococcus faecalis* is responsible for about 80% of all enterococcal infections in humans, while *Enterococcus faecium* causes only about 20% of the infections^2^. Vancomycin-resistant enterococci (VRE) were discovered in 1980^3,4^, and have become one of the most problematic multiresistant hospital associated pathogens^5^. *E*. *faecium* represents the majority of the VRE^6^.

Glycopeptide antibiotics are key drugs used in the treatment of enterococcal infections, especially those caused by *E*. *faecium*, which is typically resistant to penicillin antibiotics. Vancomycin resistance is due to the synthesis of modified cell wall precursors with decreased affinity for glycopeptides^7^. There are two vancomycin resistance gene clusters of clinical relevance: the *vanA* operon and the *vanB* operon^8^, normally carried on the transposable elements Tn1546 and Tn1549^9,10^. The resistance genes can be transferred between strains by plasmids and through conjugative transposons. Globally, the *vanA* gene cluster is the most prevalent, and it is predominantly carried by *E*. *faecium*^11^.

VRE infections are increasingly common, difficult to treat and often appear as part of long-lasting outbreaks in health care institutions, presenting tremendous challenges for infection control^12^. Apart from individual outbreaks^13^, vancomycin resistance in enterococci is still rare in Norway compared to countries in eastern and southern Europe^14,15^ and the USA, where a high percentage of resistance is reported^16^. In Europe, the mean rate of vancomycin resistance in invasive *E*. *faecium* (VREfm) in 2016 was 11.8% with a very high geographic variation^14^. In Norway, both infection and colonization with VRE are notifiable conditions. Until 2010, the number of reported VRE cases was low, with an average of only four cases each year^17^. In 2010, a major VRE outbreak was discovered at Haukeland University Hospital on the west coast of Norway, leading to 289 reported cases of VRE infection or colonization. This outbreak was caused by *E*. *faecium* with the *vanB* resistance gene^18^.

The outbreak at Østfold Hospital Trust described in this study (the SØ12 outbreak) began in August 2012, and is to date the second largest VRE-outbreak recorded in a Norwegian hospital. During the five year-period prior to outbreak, only two VRE isolates were reported from the county of Østfold, both of those in 2011.

In retrospect, we analyzed the VRE isolates identified in the course of the outbreak by using Pulsed Field Gel Electrophoresis (PFGE), often considered the gold standard profiling method for epidemiological investigations of bacterial outbreaks^19^, and MALDI-TOF mass spectrometry (MS), as well as Single Nucleotide Polymorphism (SNP). The aim of the study was twofold. The first aim was, based on the PFGE results, to understand the dynamics at different stages of this low prevalence outbreak and evaluate the infection control measures that were taken. The second aim of the study was to evaluate whether typing and clustering based on results from the simpler and faster SNP and MALDI-TOF methods are comparable to those obtained when using PFGE, thus providing a more immediate information channel in future outbreaks.

## Materials and Methods

### Ethics statement

The study was registered as a quality control project at the Norwegian Centre for Research Data (project number 39871). A request of assessment was sent to the Regional Committees for Medical and Health Research Ethics, and a full application was not considered necessary.

### Outbreak patient population and infection control measures

The SØ12 outbreak was discovered when a *vanA* VREfm was isolated from the wound of a patient with a surgical site infection. The patient was hospitalized in one of the hospital surgery wards. This led to screening of all inpatients on this ward. In this initial screening, three more VRE-positive patients were found and a nosocomial outbreak of VRE was thus recognized. In order to contain the outbreak, interventions and enhanced infection control policies were implemented immediately in all hospital wards. In departments where antibiotic pressure was considered high (e.g. department of infectious diseases, intensive care units), all inpatients were tested for VRE.

Throughout the following year, screening of all inpatients was repeated at regular intervals, in order to discover if the outbreak had spread to new bed units. To avoid further spread of VRE in the hospital, all VRE-positive patients were isolated with contact precautions. The medical records were labeled so that patients colonized with VRE could be recognized on future admissions.

The importance of hand hygiene was emphasized, and the number of available dispensers for hand disinfection was increased. In the outbreak wards, cleaning in general, and point disinfection in particular, were reinforced, and hospitalization of patients in the ward corridor was no longer accepted. Patients who had been admitted to the hospital while the spread of VRE was assumed to be at the highest (July–September 2012) were screened if rehospitalized. Each new VRE case led to further screening of all patients on the ward in which the patient was hospitalized. Prior to the outbreak, the hospital already cooperated well with the municipal health services and this was very useful in the outbreak setting. Patients were screened before transfer between the hospital and primary health care institutions. To discover satellite outbreaks, thorough screenings were also conducted in nursing homes where residents were VRE-positive. VRE-sampling was also performed ahead of transfer to other hospitals, and with readmission of patients who had previously stayed at the hospital during the outbreak period. In accordance with recommendations from the Norwegian Institute of Public Health, hospital staff was not tested^20^.

From October, the number of new cases was declining steadily, from eight cases in November 2012 to one case per month in May and June 2013. In July and August 2013 all patients on the major wards were screened, and no more cases were detected. The outbreak was considered over after the last screening in August.

### VRE laboratory screening

VRE sampling was conducted using rectal swabs (Amies agar gel, Copan, Murrieta, USA) that were subsequently inoculated directly onto a chromogenic agar (VRE CHROMagar, SmithMed, Ås, Norway) and incubated for 48 hours. Identification was done by using the rapidID 32 strep system (bioMerieux, La Balme les Grottes, France), and in addition PYR-, LAP-, and sulfa disk results were recorded.

The vancomycin minimum inhibitory concentration (MIC) was determined using E-test (bioMerieux, La Balme les Grottes, France), and interpreted according to the clinical breakpoints by the European Committee on Antimicrobial Susceptibility Testing (EUCAST). Resistance was confirmed by detection of the *vanA* resistance gene by PCR analysis^21^.

In addition to culture, a vanA/vanB VRE PCR (Xpert vanA/vanB Cepheid, Sunnyvale, USA) was performed directly on sample material (i.e. without prior culture) in situations when a rapid result was particularly important. In most cases this PCR test was used before transfer of patients to other health care institutions, but to confirm VRE carriage a positive result from culture was also required.

All isolates were stored at −70 °C, and genotyping analyses were performed retrospectively.

### Pulsed-Field gel electrophoresis

PFGE was performed based on the procedure of Murray *et al*.^22^ and the improved protocol from Turabelidze *et al*.^23^. Some modifications were made to achieve optimal results. Cultures identified as vancomycin resistant *E*. *faecium* were harvested, washed with cell suspension buffer (10 mM Tris-HCl and 1 M NaCl), and 1 mg/ml lysozyme and 0.04 Units/ml mutanolysin were added immediately before 1:1 addition of 2% low-melt pulsed-field agarose (Bio-Rad, Hercules, USA). An aliquot of the mixture was transferred to a plug-mold and left to set. To achieve optimal cell-lysis, lysis was performed for 1–3 hours at 37 °C. The plugs were washed twice in dH_2_O, proteolysed at 50 °C overnight, and washed 5 times in TE-buffer (10 mM Tris-HCl, 0.1 mM EDTA). Digestion with *SmaI* (Promega Corporation, Madison, USA) was performed for minimum 4 hours at 25 °C or overnight at room temperature. Electrophoresis was performed using the CHEF-DR III apparatus from Bio-Rad Laboratories. The gel was stained for 30–90 minutes with SYBR-gold Nucleic acid Gel Stain (Thermo-Fisher Scientific, Waltham, USA), and visualized on GelDoc EZ Imager and blue tray (Bio-Rad). The band pattern was interpreted by visual inspection using established criteria from Tenover *et al*.^24^, and analysed by BioNumerics 7 (Applied Maths NV, Sint-Martens Latem, Belgium) with Dice Coefficient. It was represented by unweighted pair group method with algorithmic mean (UPGMA) with 0.75% optimization and 1% tolerance.

For quality control and standardization between experiments the *vanB* positive *E*. *faecium* V583 was plugged, cut and analysed in parallel with up to 12 additional isolates on PFGE.

### SNP genotyping

Based on an MultiLocus Sequence Typing (MLST) database^25^, a single nucleotide polymorphism (SNP) based genotyping method using informative SNPs and an allele specific real-time PCR methodology, has been developed to study the population structure of clinical *E*. *faecium* and *E*. *faecalis*^26^.

VRE isolates were cultivated on blood agar (Oxoid Ltd., Hampshire, UK) and further sub-cultured in Brain Heart Infusion (BHI) broth (Oxoid). Total DNA was extracted using the DNeasy® Blood and Tissue kit according to the protocol for isolation from Gram-positive cells (Qiagen, Venlo, Netherlands). The previously developed highly-discriminatory SNP genotyping method was performed for profiling the isolates using eight allele-specific real-time PCRs^26^. Primers are listed in Supplementary Information Table 1. Each reaction was performed in 25 µl containing 12.5 µl 2x QuantiTect SYBR Green PCR mastermix (Qiagen), 0.3 µM of each primer (Thermo-Fisher Scientific) and approximately 200 ng genomic DNA. Cycling was performed on the AriaMX Real-Time PCR system (Agilent Technologies, Santa Clara, CA, USA) as previously described^26^, and isolate-specific SNP profiles of VRE isolates were generated based on the polymorphism presented at each SNP. Sanger sequencing was used for SNP confirmation for a few cases where the nucleotide polymorphism in the 3′end of the primers yielded ΔC_T_ < 3 between matched and unmatched primer in the real-time PCR, defined as the threshold for identification of SNP site.

### MALDI-TOF MS

The isolates were cultivated on blood agar overnight, and an ethanol/formic-acid extraction procedure was performed according to the manufacturers’ recommendations (Bruker Daltonik). MALDI-TOF MS spectra from the extraction products were obtained on a Microflex LT (Bruker Daltonics, Massachusetts, USA) and analyzed with FlexControl Software 3.0. All strains were spotted in duplicates or triplicates, and each replicate was measured three times. The resulting spectra were assembled in order to generate a mean spectrum profile (MSP) for each analyzed strain, and downloaded into the BioNumerics software version 7.5. An UPGMA dendrogram (unweighted pair group method with algorithmic mean) was created using Pearson correlation as similarity coefficient.

### Medical records and epidemiological analyses

As part of the measures taken during and after the outbreak, medical records of all patients with a positive finding for VRE were included in an outbreak database used to guide epidemiological investigations, including risk factors and outcomes. These data contributed to this study in terms of mapping the development of the outbreak (i.e. number of admissions, which wards and when the patients were in the hospital). Genetic characterization of the isolates by the PFGE was used for epidemiological characterization of the outbreak.

## Results

Contact tracing and screening revealed a total of 89 cases before the outbreak came to an end. 86 patients were colonized, while only three had an infection. All isolates were *E*. *faecium* harboring the *vanA* resistance gene. Three of the SØ12 cases were identified at other hospitals, and were not available for further analysis in this study.

**Figure 1:**
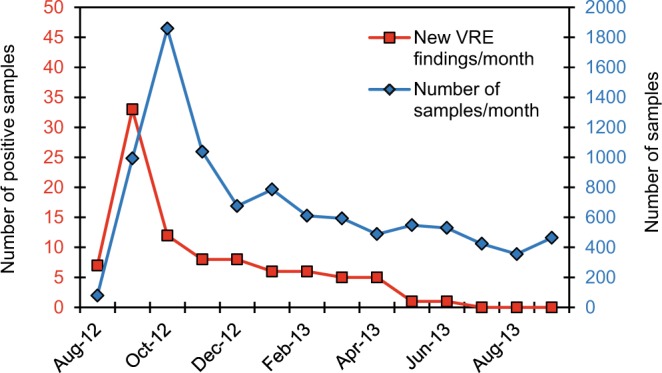
VRE screening during the SØ12 outbreak. Number of screened fecal samples (blue line) and patients positive in at least one sample (red line) during the 2012–2013 VRE outbreak at Østfold Hospital Trust. Distribution of patients positive for VRE was highest in September with 33 positive isolates. By July 2013, no more positive samples were found among the screened samples.

A total of 86 *vanA* VREfm isolates were identified as part of the SØ12 outbreak at our hospital, through screening of a total of 9454 samples (Fig. 1). Among these, 2097 Xpert vanA/vanB VRE PCR analyses were performed directly on sample material during the outbreak, leading to the detection of the *vanA* gene in 31 samples, of which 26 were confirmed by culture. The negative predictive value of the *vanA* part of the PCR test was 99.95%. The *vanB* gene PCR was positive in 1077 of the samples, but no *vanB* VREs were isolated by culture. Hence, the negative predictive value of the *vanB* part of the test was 100%, but the specificity was only 48.6%.

All 86 outbreak isolates were phenotypically and genotypically resistant to glycopeptide antibiotics. The MIC values for vancomycin for all isolates were ≥256 µg/mL. All isolates were also resistant to ampicillin.

### Clonal analysis by PFGE

**Figure 2:**
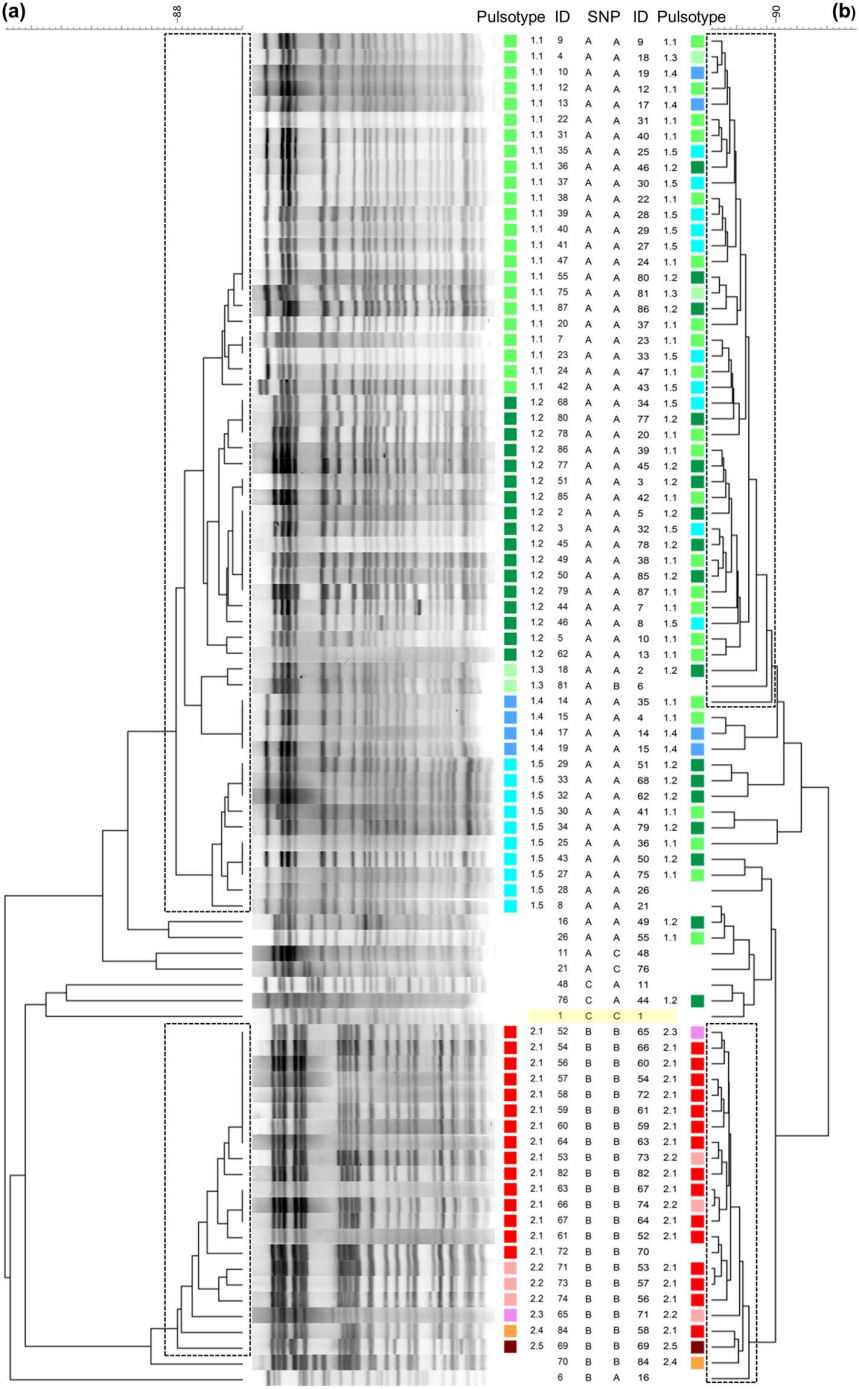
Clustering of the SØ12 VRE isolates. (**a**) Dendrogram of *SmaI* macro-restriction pattern resolved in PFGE of VRE *E*. *faecium* with *vanA*. The UPGMA tree illustrates the distance between isolates. The two boxes show the two main clusters of isolates displaying >88% similarity, while the pulsotypes defined with >94% similarity and visual inspection are differentiated with colors and number. Pulsotype 1.1–1.5 (green/blue) belongs to cluster 1, while pulsotype 2.1–2.5 (red/pink/brown) belongs to cluster 2. The index case strain (ID 1) is highlighted with a yellow box. **(b)** Dendrogram of the same isolates of VREfm resolved with MALDI-TOF MS. The two boxes indicate the two main clusters identified with >90% similarity. The pulsotypes are assigned by PFGE in (**a**).

The PFGE pattern of *smaI* restricted chromosomal DNA from the 86 VRE isolates revealed two main clusters using a similarity cut-off >88% (Fig. 2a), indicating that two genetically different strains of *E*. *faecium* caused two overlapping outbreaks. Seventy-seven of the VRE isolates belonged to one of these two clusters, while the remaining VRE isolates (n = 9, 10.5%), including the presumptive index case isolate, showed heterogeneous and unique PFGE patterns and were probably not related to any of the two main clusters, i.e. these were likely sporadic cases.

The largest cluster (cluster 1) consisted of 56 isolates (65.1%) and contained all strains isolated during the first 12 weeks of the outbreak, except for seven sporadic strains. By visual inspection and a similarity cutoff >94% this cluster was further divided into five pulsotypes (1.1–1.5). The predominant pulsotype (1.1) comprised 23 isolates (26.7%). The second most dominant pulsotype (1.2) comprised 17 isolates (19.8%). Several isolates were indistinguishable from each other and hence clonally identical.

**Figure 3:**
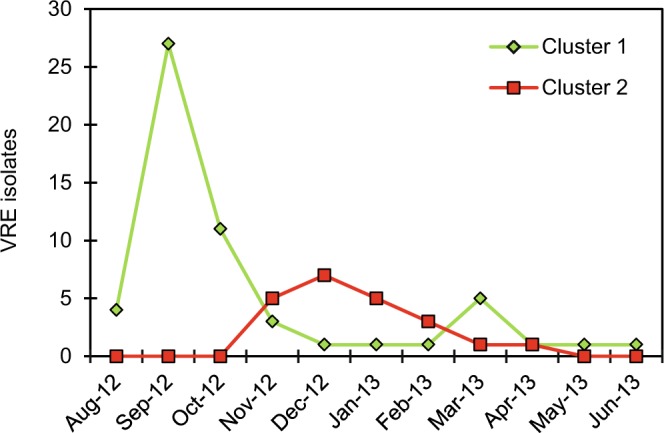
Monthly distribution of cluster 1 and cluster 2 related strains. Strains belonging to cluster 1 (green line) and cluster 2 (red line) are defined by >88% similarity by PFGE during the outbreak period. Strains unrelated to the two main clusters are not included in the chart. Most of the isolates in cluster 1 were found during screening September and October 2012, concurrent with the decline of positive isolates related to strains in cluster 1, cluster 2 related strains were introduced in November 2012.

The second cluster (cluster 2) showed a distinct pattern, compared to cluster 1, and consisted of 21 isolates (24.4%), corresponded to a second strain which was introduced at a later time point during the outbreak (Fig. 3). By visual inspection and a similarity cut-off >94% this second group was further divided into five pulsotypes (2.1–2.5). The predominant pulsotype in the second group (2.1) comprised 15 isolates (17.4%).

### MALDI-TOF MS typing

**Figure 4:**
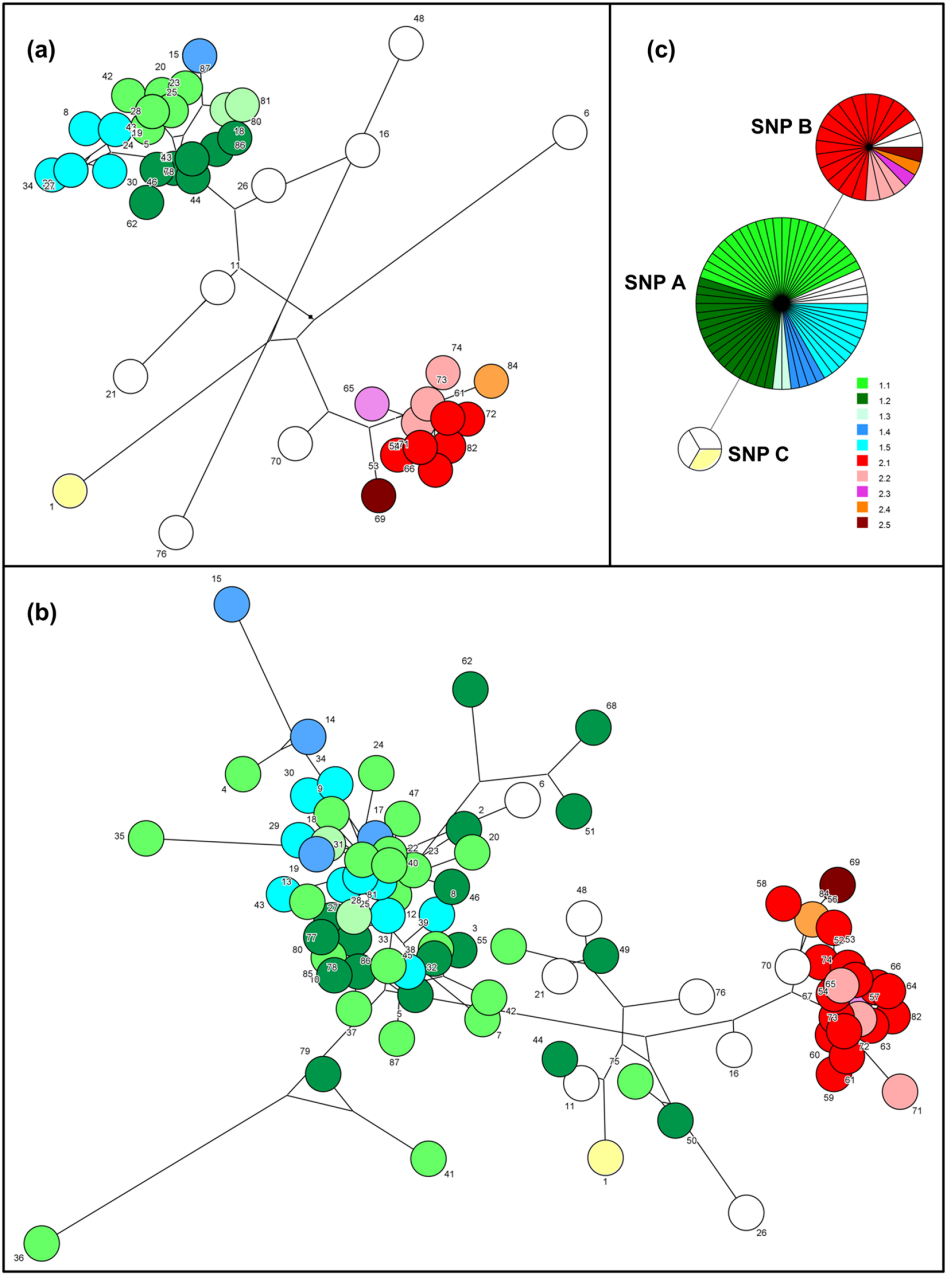
Minimum spanning trees demonstrating the relatedness among SØ12 *E*. *faecium* isolates. The network length between the isolates based on cluster analysis of the **(a)** PFGE, **(b)** MALDI-TOF and **(c)** SNP results are shown. Pulsotypes are indicated with colors; green and blue correspond to isolates in cluster 1, while red and pink correspond to isolates in cluster 2. Unrelated strains are illustrated colorless, except for the index case strain in yellow color. For PFGE, the total network length is 447, and for MALDI-TOF the length is 358. The SNP results are viewed as minimum spanning tree (MST).

Identification was confirmed by MALDI-TOF MS after the outbreak had ended. All isolates were identified as *E*. *faecium* with a log (score) ≥2.3, which is above the score value 2.0 needed for species identification according to the manufacturer. Analysis of the MSP spectra divided the isolates into two clusters, with >90% similarity in each, as shown in Figs 2b and 4. The initial outbreak cluster contained 43 isolates (50%), while 23 isolates belonged to the second cluster (26.7%) and 20 isolates (23.3%) did not belong to any of the two main clusters using 90% similarity as cut-off. All isolates assigned to cluster 1 or 2 by MALDI-TOF were also assigned to the corresponding cluster 1 and 2, respectively, by PFGE, with three exceptions. One strain found unrelated to the two outbreak clusters with PFGE was placed in cluster 1 by MALDI-TOF and two other unrelated strains were placed in MALDI-TOF cluster 2. Furthermore, MALDI-TOF identified less isolates belonging to cluster 1 than PFGE; 43 vs 53 isolates. A total of 20 vs nine isolates were not classified to either of the two main clusters by MALDI-TOF compared to PFGE.

Using Pearson Correlation the congruence between PFGE and MALDI-TOF was found to be 63.2% and the correlation (r) was strong (72.4%). However, regarding further sub-clustering within the two main clusters, the congruence between PFGE and MALDI-TOF was only 3.0% and 26.3% and the correlation was low (17.1%) and medium (48.5%) for cluster 1 and 2, respectively (Fig. 2).

### SNP polymorphism

Genotyping of the 86 isolates using eight allele-specific primers and real-time PCR revealed three SNP profiles among the isolates (A-C listed in Table 1 and shown in Fig. 2). The dominant profile GGCAGACC (profile A) was shared by 60 isolates in our collection (69.8%), including the 56 isolates belonging to the PFGE outbreak cluster 1 (Fig. 2). The SNP profile GGTAGGCC (profile B) was shared by 23 isolates (27.7%), including all strains from the PFGE cluster 2. Three isolates (3.5%) including the index case isolate and two unrelated strains shared the SNP profile GATAGATC (profile C). For 20 isolates, polymorphisms present in the gyd160 SNP site showed a ∆C_T_ value below the set threshold between matched and mismatched primer, and these sites were therefore also determined using Sanger sequencing. Moreover, the SNP site pstS87 was confirmed by Sanger sequencing for two isolates, and SNP site atpA188 for another two isolates.

**Table 1:** SNP site polymorphism used for genotyping of *E*. *faecium*^26^.

	SNP Profile A	SNP Profile B	SNP Profile C
pstS452	G	G	G
atpA485	G	G	A
gyd160	C	T	T
purK115	A	A	A
pstS87	G	G	G
atpA314	A	G	A
atpA188	C	C	T
purK217	C	C	C
No of isolates	60	24	3

### Medical records and epidemiological findings

Cases were reported from more than ten different wards out of a total of 23, including both medical and surgical specialties. Retrospective review of hospital admissions showed that many patients were admitted to multiple wards during their hospital stays. VRE-positive patients had in general more hospital admissions (in average 3 admissions of positive patients vs 1 admission of negative patients), and longer exposure (mean = 12 days for VRE-positive patients vs 6 days for VRE negative patients). A total of 40 VRE-carrying patients were identified in the first two months of the outbreak (August and September 2012).

The majority of patients from VRE cluster 1 (44 out of 56 patients) were hospitalized in July, August or September 2012 (Fig. 3) in one of the following three hospital wards, referred to as the index outbreak wards: Gastric surgery, Infectious diseases, and Pulmonary diseases. Seven of the remaining 12 patients were indirectly associated with one of these three hospital wards. They had been hospitalized together with a VRE-positive patient with previous stay at one of these wards during July-October, however at the time the VRE-positive diagnosis was not yet stated. In particular, in March 2013, four isolates with pulsotype 1.2 (ID 77–80) were identified (Fig. 2), even though this pulsotype, or any isolate belonging to cluster 1, had not been found since January 2013. Review of their medical records showed that patients numbered 78–80 were hospitalized simultaneously in March at the same ward, and that patient number 78 had previously been hospitalized at one of the three index wards on two occasions in July 2012, before the primary outbreak was discovered.

None of the 21 isolates belonging to cluster 2 were isolated from patients that had been admitted to any of the index outbreak wards in the three first months of the outbreak. Before proven positive, 15 of these patients had tested negative in earlier screening tests conducted after the peak of the outbreak.

## Discussion

Spread of VRE is a serious issue in many hospitals. Rapid and sensitive microbial detection is important to limit the spread of infection, and determination of epidemiological relatedness is necessary for investigation of contamination patterns of resistant bacteria, and for outbreak management. Genotyping results are, however, often available only far into the outbreak, or even after the situation has ended.

In this study, we describe epidemiological relatedness of Vancomycin-resistant *E*. *faecium* isolated during the SØ12 outbreak at a Norwegian hospital in 2012–2013. We first aimed to consider the infection control measures in light of the PFGE-based genetic characterization, which revealed that the outbreak consisted of two large clusters of strains with >88% similarity (Fig. 3), rather than just one as anticipated.

From screening conducted throughout all hospital wards, the majority of patients with VRE strains in PFGE cluster 1 were hospitalized within the three consecutive months July, August and September 2012, in only three of the 23 hospital wards (the three index wards). In addition, a few isolates belonging to cluster 1 were detected in patients hospitalized together in March 2013, of which one had previously been hospitalized in one of the index wards July 2012. Likely, this patient carried the infection at that time, and initiated the small outbreak when rehospitalized March 2013. In conclusion, spread of isolates belonging to cluster 1 seems to mainly have taken place in three hospital wards (Gastric surgery, Infectious diseases, and Pulmonary diseases) during a three-month period starting from July 2012, before the outbreak was recognized. After the discovery of the first VRE isolate mid-August 2012, 49 isolates were identified by the end of October, 42 belonging to cluster 1 and seven sporadic cases. Following initiation of infection control measures throughout September, a continuous and rapid decreasing incidence could be seen, proving that the increased focus on infection control and the measures taken were highly effective in limiting the outbreak. The importance of hand hygiene and other infection control issues in limiting the spread of hospital bacteria is also well documented in previous studies^27–30^.

A distinct VRE strain introduced in November 2012 gave rise to a second outbreak (PFGE cluster 2), just as the initial outbreak was declining. The majority of isolates belonging to cluster 2 were from patients who had not been hospitalized in any of the three index outbreak wards in the three summer months of 2012, and most of these patients had screened negative in the early phase of the outbreak. This second cluster primarily affected other hospital wards than those affected in the initial part of the SØ12 outbreak. The better understanding of the outbreak dynamic obtained with PFGE genotyping results underscores the great importance of the implemented infection control measures. The fact that a new cluster was identified makes it less likely that the last part of the SØ12 outbreak was due to relocation of patients or failure of preventive strategies, which was a reasonable hypothesis before the genotyping results were available. The second outbreak did not spread as much as the first, probably because effective infection control measures were already established.

An interesting finding in this study is the fact that the first (index case) isolate, which was a clinical isolate and the reason why screening was initiated, was unrelated to any of the two outbreak clusters, and hence not actually linked to the large outbreak in the three index wards caused by cluster 1 VRE strains. Thus the outbreak was discovered by coincidence, which underscores the need for systematic screening of potentially at-risk patients, including all patients transferred from hospitals abroad or from central large hospitals, for early detection of hospital outbreaks.

As is common in outbreaks with VRE, the majority of VRE-positive patients in our study was colonized with the bacteria, but did not have an infection. The strains found unrelated to both cluster 1 and 2, including the index case isolate, may have originated from patients colonized with VRE prior to the outbreak. It is unknown how long VRE is carried, but colonization could persist from weeks to months^31–33^, hence colonized patients will occasionally be admitted to hospitals and other health care institutions. However, three of the nine unrelated isolates were found in patients who had screened negative for VRE in an early phase of the outbreak, and thus the place and time for their colonization remains unknown.

In our outbreak setting, we could successfully use a vanA/vanB PCR method for rapid identification of *vanA*-VRE, as part of the outbreak control strategy. The negative predictive value of the test was excellent, and isolation precautions could therefore be terminated when a negative test result was recorded. The method had, however, a poor specificity and a high false positive rate for the *vanB* gene, also described by others^34,35^. The reason is most likely the frequent occurrence of this gene in various other bacterial species in the human gut. Despite this, the Cepheid Xpert vanA/vanB PCR has previously been reported useful in preventing the spread of an epidemic *vanA/vanB E*. *faecium* strain in a French hospital^36^.

A second major aim of this study was to assess whether simpler and faster methods, like SNP profiling or MALDI-TOF MS, could prove useful in routine monitoring of strains isolated in an outbreak situation, rather than the time-consuming PFGE.

By the use of eight informative SNP sites, SNP profiling was previously shown to be a relatively rapid, cost-effective and good alternative to MLST characterization of *E*. *faecium* isolates^26^. In our study, we found that all isolates belonging to PFGE cluster 1 shared the SNP profile A, while all isolates belonging to PFGE cluster 2 shared the SNP profile B. Three of the nine strains unrelated to PFGE clusters 1 or 2, shared a third distinct SNP profile, including the index strain, while the remaining strains had either A or B SNP profiles. Due to the low resolution within the two main clusters, and the inconsistent distribution between strains unrelated to the two main clusters, the method will have limitations in small local epidemiological studies, with limited allelic variation between isolates. Since real-time PCR technology is available in most routine laboratories, the method could, however, serve as a good choice for long-term studies of VRE isolates or for international comparison studies between hospitals, where the allelic variations at the SNP sites are greater. Furthermore, high-resolution melting (HRM) analysis could be used as an additional and parallel typing method to SNP specific real-time PCR. HRM analysis incorporates not only information from each key SNP, but also from neighboring SNPs, and this information can improve the discriminatory power^37^.

MALDI-TOF MS has emerged as a fast, easy and low-cost identification method for clinical pathogens. In addition, the method is promising for antibiotic resistance detection^38^, and is shown to be able to distinguish between *vanB* resistant and sensitive *E*. *faecium* isolates^39^. Recent advances in both sample preparation and data analysis have increased the methods’ resolution and potential for subspecies level classification of several emerging pathogens^40–43^. MALDI-TOF MS has previously been found to differentiate patterns of hospital associated strains compared to other strains, but failed in identifying lineage specific markers in *E*. *faecium*^44,45^. In this study, we demonstrated that MALDI-TOF MS is a good alternative to PFGE when it comes to time consumption and rapid screening, showing an overall strong correlation with PFGE (r = 72.4%). The two methods identified both outbreak clusters, but for cluster 1, only 76% of the isolates were correctly assigned with MALDI-TOF, and even some strains found clonally identical with PFGE were shown to be less than 90% identical with MALDI-TOF. Moreover, within each cluster, only a low or medium correlation between the two methods was found. Altogether, these results show a lower discriminating power of MALDI-TOF compared to PFGE at the strain level for *E*. *faecium*. It is however important to take into account that different typing techniques measure different cellular properties^46^. The strain specificity of MALDI-TOF MS is confined since mainly conserved ribosomal proteins are detected^47^, and evolutionary DNA changes observable with genotypic typing techniques are less likely detected^46^. Discrimination at the strain level is influenced by minor changes in the mass spectra, which highlights the importance of applying similar culture conditions (i.e. time, growth temperature, medium) and sample preparation methods to obtain good spectrum quality and reproducibility^48^.

The major advantage with MALDI-TOF MS is its wide use as a routine microbial identification technique in hospitals worldwide, and typing information is thus readily available during an outbreak. Studies have suggested that the method could serve as a first-line typing tool for investigation of possible hospital outbreaks of microorganisms such as *E*. *faecium* and *Staphylococcus aureus*^49,50^, but optimized and reproducible protocols and databases are needed. Based on our findings, although the taxonomic resolution is lower than for PFGE, readily available MALDI-TOF results may be of high value for real-time outbreak detection and management. Even if MALDI-TOF MS data are not optimal for accurate retrospective epidemiological typing, they could prove useful in routine infection surveillance and control strategies.

Although PFGE is a cumbersome method, it has up to recently been considered the gold-standard in epidemiological investigation of hospital outbreak situations due to the high resolution and ability to discriminate strains with minor genetic changes^51^. This is especially important in typing hospital associated strains where the rate of recombination is assumed to be relatively low compared to the high rate of recombination found elsewhere^52,53^. In our investigation of the SØ12 outbreak, PFGE showed, as expected, and above the SNP profiling and MALDI-TOF MS analysis, the best resolution. However, results from all three methods suggested that the SØ12 outbreak actually embraced two different outbreaks originating from two distinct *E*. *faecium* strains. In addition, all three methods identified the index case strain as unrelated to the two main outbreak strains. The major drawback with PFGE is the time consuming laboratory protocol which may hamper or delay the discovery of an outbreak. The DNA restriction patterns may differ slightly due to technicalities and technician performances, and even if the band pattern analysis is performed by data software, subjective investigation by trained personnel is often necessary. Moreover, single variations can occur at restriction sites and lead to more than one band shift which may affect the epidemiological investigation^19^.

Whole genome sequencing (WGS) of bacterial pathogens via Next Generation Sequencing (NGS) technologies has emerged as a powerful tool for determining the relatedness of bacterial isolates in outbreak situations^54–56^. WGS analysis of entire genomes provides markedly higher resolution than those of conventional methods, and full genetic information can be obtained. Investigations of relatedness can be based on entire genome sequences, or e.g. *in silico* MLST or various SNP analyses can be performed. The SNP profiling done in the present work was based on eight informative SNP sites^26^, but several other SNP sites would be of great value for more accurate typing. Powerful NGS pipelines with reference genomes, e.g. the high-accuracy pipeline BactSNP^57^, are available for reliable epidemiological typing. NGS based methods have previously been shown to yield the best resolution and accurate epidemiological concordance of outbreaks with VRE^54,58^, and could certainly be used to further investigate the SØ12 outbreak. While WGS show many advantages over standard microbiological methods, it is not yet widely implemented in routine hospital diagnostics. Notable challenges have included bioinformatics workflow, costs, manpower, laboratory infrastructure, and quality control, but the technology is continuously developing towards more simple and affordable solutions^59,60^.

Taken together, our present retrospect analysis of VRE isolates by PFGE allowed us to characterize the dynamics of the SØ12 outbreak, which surprisingly consisted of two distinct outbreaks. Moreover, our comparison of genotyping and clustering methods showed that despite lower resolution than PFGE, readily available MALDI-TOF MS data may be of great importance for infection control and prevention if routinely used in real-time microbiological surveillance in health care institutions.

## Supplementary information

Primers used for SNP-profiling of E.faecium.

## Data Availability

The experimental data that support the findings of this study are available from the corresponding author, A.R, upon reasonable request. Medical Records that support the epidemiological finding of this study are not publicly available due to privacy restrictions.

## References

[1] Fisher K, Phillips C (2009). The ecology, epidemiology and virulence of Enterococcus. Microbiology..

[2] Hidron AI (2008). NHSN annual update: antimicrobial-resistant pathogens associated with healthcare-associated infections: annual summary of data reported to the National Healthcare Safety Network at the Centers for Disease Control and Prevention, 2006–2007. Infec Control Hosp Epidemiol..

[3] Leclercq R, Derlot E, Duval J, Courvalin P (1988). Plasmid-mediated resistance to vancomycin and teicoplanin in Enterococcus faecium. N Engl J Med..

[4] Sahm DF (1989). *In vitro* susceptibility studies of vancomycin-resistant Enterococcus faecalis. Antimicrob Agents Chemother..

[5] Cattoir V, Leclercq R (2013). Twenty-five years of shared life with vancomycin-resistant enterococci: is it time to divorce?. J Antimicrob Chemother..

[6] Sievert DM (2013). Antimicrobial-resistant pathogens associated with healthcare-associated infections: summary of data reported to the National Healthcare Safety Network at the Centers for Disease Control and Prevention, 2009–2010. Infect Control Hosp Epidemiol..

[7] Walsh C (2000). Molecular mechanisms that confer antibacterial drug resistance. Nature..

[8] Hollenbeck BL, Rice LB (2012). Intrinsic and acquired resistance mechanisms in enterococcus. Virulence..

[9] Arthur M, Molinas C, Depardieu F, Courvalin P (1993). Characterization of Tn1546, a Tn3-related transposon conferring glycopeptide resistance by synthesis of depsipeptide peptidoglycan precursors in Enterococcus faecium BM4147. J Bacteriol..

[10] Wardal E (2014). Molecular analysis of vanA outbreak of Enterococcus faecium in two Warsaw hospitals: the importance of mobile genetic elements. Biomed Res Int..

[11] Hammerum AM (2017). Emergence of vanA Enterococcus faecium in Denmark, 2005-15. J Antimicrob Chemother..

[12] Miller WR, Murray BE, Rice LB, Arias CA (2016). Vancomycin-Resistant Enterococci: Therapeutic Challenges in the 21st Century. Infect Dis Clin North Am..

[13] Pinholt M (2015). Multiple hospital outbreaks of vanA Enterococcus faecium in Denmark, 2012-13, investigated by WGS, MLST and PFGE. J Antimicrob Chemother..

[14] ECDC. Surveillance of antimicrobial resistance in Europe Annual Report of the European Antimicrobial Resistance Surveillance Network (EARS-Net). *European Centre for Disease Prevention and Control*. (2016).

[15] Franyó Dorottya, Kocsi Balázs, Lesinszki Virág, Pászti Judit, Kozák Anita, Bukta Evelin Erzsébet, Szabó Judit, Dombrádi Zsuzsanna (2018). Characterization of Clinical Vancomycin-Resistant Enterococcus faecium Isolated in Eastern Hungary. Microbial Drug Resistance.

[16] Reik R, Tenover FC, Klein E, McDonald LC (2008). The burden of vancomycin-resistant enterococcal infections in US hospitals, 2003 to 2004. Diagn Microbiol Infect Dis..

[17] NIPH. *Norwegian Institute of Public Health*. Antibiotic resistance in Norway, https://www.fhi.no/en/op/hin/health–disease/antibiotic-resistance-in-norway-p/#vancomycinresistant-enterococci-vre/ (2017).

[18] NIPH. *Norwegian Institute of Public Health*. Enterokokkinfeksjon - veileder for helsepersonell (norwgian only), https://www.fhi.no/nettpub/smittevernveilederen/sykdommer-a-a/enterokokkinfeksjon-inkl.-vankomyci/ (2017).

[19] Lopez-Canovas L, Martinez Benitez MB, Herrera Isidron JA, Flores Soto E (2019). Pulsed Field Gel Electrophoresis: Past, present, and future. Anal Biochem..

[20] NIPH. *Norwegian Institute of Public Health*. Håndtering av vankomycinresistente enterokokker (VRE) ved norske sykehus og sykehjem (norwegian only), https://www.fhi.no/sv/forebygging-i-helsetjenesten/smittevern_i_institusjoner/tiltak/handtering-av-vankomycinresistente-/#proevetaking-for-vre-i-sykehus-og-sykehjem/ (2010).

[21] Palladino S, Kay ID, Costa AM, Lambert EJ, Flexman JP (2003). Real-time PCR for the rapid detection of vanA and vanB genes. Diagn Microbiol Infect Dis..

[22] Murray BE, Singh KV, Heath JD, Sharma BR, Weinstock GM (1990). Comparison of genomic DNAs of different enterococcal isolates using restriction endonucleases with infrequent recognition sites. J Clin Microbiol..

[23] Turabelidze D, Kotetishvili M, Kreger A, Morris JG, Sulakvelidze A (2000). Improved pulsed-field gel electrophoresis for typing vancomycin-resistant enterococci. J Clin Microbiol..

[24] Tenover FC (1995). Interpreting chromosomal DNA restriction patterns produced by pulsed-field gel electrophoresis: criteria for bacterial strain typing. J Clin Microbiol..

[25] Homan WL (2002). Multilocus sequence typing scheme for Enterococcus faecium. J Clin Microbiol..

[26] Rathnayake IU, Hargreaves M, Huygens F (2011). Genotyping of Enterococcus faecalis and Enterococcus faecium isolates by use of a set of eight single nucleotide polymorphisms. J Clin Microbiol..

[27] Weber DJ, Anderson D, Rutala WA (2013). The role of the surface environment in healthcare-associated infections. Curr Opin Infect Dis..

[28] Eckstein BC (2007). Reduction of Clostridium Difficile and vancomycin-resistant Enterococcus contamination of environmental surfaces after an intervention to improve cleaning methods. BMC Infect Dis..

[29] Ulrich N, Vonberg RP, Gastmeier P (2017). Outbreaks caused by vancomycin-resistant Enterococcus faecium in hematology and oncology departments: A systematic review. Heliyon..

[30] Kampmeier S (2017). Weekly screening supports terminating nosocomial transmissions of vancomycin-resistant enterococci on an oncologic ward - a retrospective analysis. Antimicrob Resist Infect Control..

[31] Whelton E (2016). Vancomycin-resistant enterococci carriage in an acute Irish hospital. J Hosp Infect..

[32] Shenoy ES, Paras ML, Noubary F, Walensky RP, Hooper DC (2014). Natural history of colonization with methicillin-resistant Staphylococcus aureus (MRSA) and vancomycin-resistant Enterococcus (VRE): a systematic review. BMC Infect Dis..

[33] Sohn KM (2013). Duration of colonization and risk factors for prolonged carriage of vancomycin-resistant enterococci after discharge from the hospital. Int J Infect Dis..

[34] Bourdon N (2010). Rapid detection of vancomycin-resistant enterococci from rectal swabs by the Cepheid Xpert vanA/vanB assay. Diagn Microbiol Infect Dis..

[35] Babady NE, Gilhuley K, Cianciminio-Bordelon D, Tang YW (2012). Performance characteristics of the Cepheid Xpert vanA assay for rapid identification of patients at high risk for carriage of vancomycin-resistant Enterococci. J Clin Microbiol..

[36] Marcade G (2014). Outbreak in a haematology unit involving an unusual strain of glycopeptide-resistant Enterococcus faecium carrying both vanA and vanB genes. The Journal of antimicrobial chemotherapy.

[37] Tong SY (2011). High-resolution melting genotyping of Enterococcus faecium based on multilocus sequence typing derived single nucleotide polymorphisms. PLoS One..

[38] Kostrzewa M, Sparbier K, Maier T, Schubert S (2013). MALDI-TOF MS: an upcoming tool for rapid detection of antibiotic resistance in microorganisms. Proteomics Clin Appl..

[39] Griffin PM (2012). Use of matrix-assisted laser desorption ionization-time of flight mass spectrometry to identify vancomycin-resistant enterococci and investigate the epidemiology of an outbreak. J Clin Microbiol..

[40] Steensels D, Deplano A, Denis O, Simon A, Verroken A (2017). MALDI-TOF MS typing of a nosocomial methicillin-resistant Staphylococcus aureus outbreak in a neonatal intensive care unit. Acta Clin Belg..

[41] Egli A (2015). Matrix-assisted laser desorption/ionization time of flight mass-spectrometry (MALDI-TOF MS) based typing of extended-spectrum beta-lactamase producing E. coli–a novel tool for real-time outbreak investigation. PLoS One..

[42] Giacometti F (2018). Application of MALDI-TOF MS for the subtyping of Arcobacter butzleri strains and comparison with their MLST and PFGE types. Int J Food Microbiol..

[43] Barbuddhe SB (2008). Rapid identification and typing of listeria species by matrix-assisted laser desorption ionization-time of flight mass spectrometry. Appl Environ Microbiol..

[44] Freitas AR (2017). Rapid detection of high-risk Enterococcus faecium clones by matrix-assisted laser desorption ionization time-of-flight mass spectrometry. Diagn Microbiol Infect Dis..

[45] Lasch P (2014). Insufficient discriminatory power of MALDI-TOF mass spectrometry for typing of Enterococcus faecium and Staphylococcus aureus isolates. J Microbiol Methods..

[46] Taneja N, Sethuraman N, Mishra A, Mohan B (2016). The 2002 Chandigarh cholera outbreak revisited: utility of MALDI-TOF as a molecular epidemiology tool. Lett Appl Microbiol..

[47] Singhal N, Kumar M, Kanaujia PK, Virdi JS (2015). MALDI-TOF mass spectrometry: an emerging technology for microbial identification and diagnosis. Front Microbiol..

[48] Goldstein JE, Zhang L, Borror CM, Rago JV, Sandrin TR (2013). Culture conditions and sample preparation methods affect spectrum quality and reproducibility during profiling of Staphylococcus aureus with matrix-assisted laser desorption/ionization time-of-flight mass spectrometry. Lett Appl Microbiol..

[49] Savas S, Hazirolan G, Karagoz A, Parlak M (2019). From days to hours: Can MALDI-TOF MS system replace both conventional and molecular typing methods with new cut off level for Vancomycin Resistant Enterococcus faecium. J Microbiol Methods..

[50] Lindgren A (2018). Development of a rapid MALDI-TOF MS based epidemiological screening method using MRSA as a model organism. Eur J Clin Microbiol Infect Dis..

[51] Goering RV (2010). Pulsed field gel electrophoresis: a review of application and interpretation in the molecular epidemiology of infectious disease. Infect Genet Evol..

[52] Willems RJ (2012). Restricted gene flow among hospital subpopulations of Enterococcus faecium. MBio..

[53] Gaca AO, Gilmore MS (2016). Killing of VRE Enterococcus faecalis by commensal strains: Evidence for evolution and accumulation of mobile elements in the absence of competition. Gut Microbes..

[54] Lytsy B, Engstrand L, Gustafsson A, Kaden R (2017). Time to review the gold standard for genotyping vancomycin-resistant enterococci in epidemiology: Comparing whole-genome sequencing with PFGE and MLST in three suspected outbreaks in Sweden during 2013-2015. Infect Genet Evol..

[55] Humphreys H, Coleman DC (2019). Contribution of whole-genome sequencing to understanding of the epidemiology and control of meticillin-resistant Staphylococcus aureus. J Hosp Infect..

[56] Tagini F, Greub G (2017). Bacterial genome sequencing in clinical microbiology: a pathogen-oriented review. Eur J Clin Microbiol Infect Dis..

[57] Yoshimura, D. *et al*. Evaluation of SNP calling methods for closely related bacterial isolates and a novel high-accuracy pipeline: BactSNP. *Microb Genom*. **5**, 10.1099/mgen.0.000261 (2019). 10.1099/mgen.0.000261 PMC6562250 31099741

[58] Schlebusch S (2017). MALDI-TOF MS meets WGS in a VRE outbreak investigation. Eur J Clin Microbiol Infect Dis..

[59] Mintzer, V., Moran-Gilad, J. & Simon-Tuval, T. Operational models and criteria for incorporating microbial whole genome sequencing in hospital microbiology - A systematic literature review. *Clin Microbiol Infect*., 10.1016/j.cmi.2019.04.019 (2019). 10.1016/j.cmi.2019.04.019 31039443

[60] Rossen JWA, Friedrich AW, Moran-Gilad J, Genomic ESGF, Molecular D (2018). Practical issues in implementing whole-genome-sequencing in routine diagnostic microbiology. Clin Microbiol Infect..

