# Supplementary material for: Molecular analysis and epidemiological typing of Vancomycin-resistant Enterococcus outbreak strains
Source: Sci Rep. 2019 Aug 15;9:11917. doi: 10.1038/s41598-019-48436-2 (PMC6695431; doi:10.1038/s41598-019-48436-2)
Supplement: Supplementary file 1 — Primers used for SNP-profiling of E.faecium. [file 41598_2019_48436_MOESM1_ESM.pdf]

Supplementary information

Molecular analysis and epidemiological typing of Vancomycin-resistant *Enterococcus* outbreak strains

Anbjørg Rangberg, Astri Lervik Larsen, Oliver Kacelnik, Hanne Skarpodde Sæther, Marthe Bjørland, Jetmund Ringstad, Christine Monceyron Jonassen

Table 1. Primers used for SNP-profiling of *E.faecium*.

Primer name	Sequence
purK115 C F	AGAAAAATCTTTTTTGGAAACGAA**C**
purK115 T F	AGAAAAATCTTTTTTGGAAACGAA**T**
purK115 R	GATCCCGTCAATCGCATCTT
purK 115 F	AGAAAAATCTTTTTTGGAAACGAA
atpA314 C F	CCGTAAAACAGGGAAAACTTC**C**
atpA314 T F	CCGTAAAACAGGGAAAACTTC**T**
atpA314 R	GATCATATCTTGACCTTTTTGGTTGA
atpA314 F	CCGTAAAACAGGGAAAACTTC
pstS452CR	GTGTACATATGTTCATATGACCAGATT**C**
pstS452TR	GTGTACATATGTTCATAKGACCAGATT**T**
pstS452F	TCGACGGTGTAGAACCAAAAGA
pstS452R	GTGTACATATGTTCATATGACCAGATT
atpA485CR	CAGCATATGGTGCGATATAAAG**C**
atpA485TR	CAGCATATGGTGCGATATAAAG**T**
atpA485F	ACATTGAAAAAATATGGCGCAAT
atpA485R	CAGCATATGGTGCGATATAAAG
gyd160GR	CCGTCTAATTTACCGTTCAATTC**G**
gyd160TR	CCGTCTAATTTACCGTTCAATTC**T**
gyd160AR	CCGTCTAATTTACCRTTCAATTC**A**
gyd160F	GCAAACATCGTWCCTAACTCAACW
gyd160R	CCGTCTAATTTACCRTTCAATTC
pstS87CF	GTGGATCATAAAGTAGCAGTGGT**C**
pstS87TF	GTGGATCATAAAGTAGCAGTRGT**T**
pstS87R	GTAAAGATATCAATCAATTCCTGTTTKG
pstS87F	GTGGATCATAAAGTAGCAGTGGT
atpA188GR	GTTAACAGATTTACGTTGCATAAC**G**
atpA188AR	GTTAACAGATTTACGTTGCATAAC**A**
atpA188F	AATYGACGGACTAGGTGAAATCG
atpA188R	GTTAACAGATTTACGTTGCATAAC
purK217AR	CCCTTGCCATCATAGCC**A**
purK217GR	CCYTTGCCATCATARCC**G**
purK217F	GATCGTCAGTCCGACRGATATC
purK217R	CCCTTGCCATCATAGCC
